# Cellular microRNA miR-26a suppresses replication of porcine reproductive and respiratory syndrome virus by activating innate antiviral immunity

**DOI:** 10.1038/srep10651

**Published:** 2015-05-27

**Authors:** Xiaojuan Jia, Yuhai Bi, Jing Li, Qing Xie, Hanchun Yang, Wenjun Liu

**Affiliations:** 1 Key Laboratory of Animal Epidemiology and Zoonosis of Ministry of Agriculture, College of Veterinary Medicine and State Key Laboratory of Agrobiotechnology, China Agricultural University, Beijing 100193, China; 2 CAS Key Laboratory of Pathogenic Microbiology and Immunology, Institute of Microbiology, Chinese Academy of Sciences, Beijing 100101, China; 3 University of Chinese Academy of Sciences, Beijing 100101, China; 4 Department of Clinical Laboratory, Beijing Shijitan Hospital, Capital Medical University, Beijing 100038, China

## Abstract

Porcine reproductive and respiratory syndrome (PRRS) has caused large economic losses in the swine industry in recent years. Current PRRS vaccines fail to effectively prevent and control this disease. Consequently, there is a need to develop new antiviral strategies. MicroRNAs play critical roles in intricate host-pathogen interaction networks, but the involvement of miRNAs during PRRS virus (PRRSV) infection is not well understood. In this study, pretreatment with miR-26a induced a significant inhibition of PRRSV replication and remission of the cytopathic effect in MARC-145 cells, and this antiviral effect was sustained for at least 120 h. Luciferase reporter analysis showed that the PRRSV genome was not the target of miRNA-26a. Instead, RNA-seq analysis demonstrated that miR-26a significantly up-regulated innate anti-viral responses, including activating the type I interferon (IFN) signaling pathway and promoting the production of IFN-stimulated genes. These findings suggest that delivery of miR-26a may provide a potential strategy for anti-PRRSV therapies.

Porcine reproductive and respiratory syndrome (PRRS) is one of the most significant viral diseases threatening the global swine industry. The causative agent is PRRS virus (PRRSV), which is a member of the order *Nidovirales*, family *Arteriviridae*^1^. PRRSV is an enveloped, single-stranded, positive-sense RNA virus with an approximately 15 kb genome containing 10 partially overlapping open reading frames (ORFs: ORF1a/1b, ORF2a/2b, ORF3-4, ORF5/5a, and ORF6-7)^1^,^2^. ORF1a and ORF1b occupy 80% of the genome and encode poly-proteins (pp1a and pp1ab) that are post-translationally processed by autoproteolytic cleavage into at least 14 nonstructural proteins (nsp1α/1β, nsp2-6, nsp7α/7β, and nsp8-12)^1^,^3^. The other ORFs are translated into structural proteins (Gp2a/E, Gp3, Gp4, Gp5/Gp5a, M and N)^1^,^2^. In recent years, the highly pathogenic PRRSV (HP-PRRSV) has resulted in enormous economic losses in China and other Southeast Asian countries^4^,^5^. However, current antiviral strategies do not effectively prevent or control HP-PRRSV. Hence, it is imperative to develop a safe and effective antiviral strategy to combat HP-PRRSV infection.

MicroRNAs (miRNAs) are small RNA molecules (~23 nt) that modulate gene expression by binding to target mRNAs and causing target cleavage or translational blockage^6^,^7^. miRNAs are involved in the cross-talk between the host and virus. Several reports show that cellular miRNAs can regulate viral infections by targeting viral genes^8^,^9^,^10^,^11^,^12^ or host genes^13^,^14^,^15^. Among these miRNAs, miR-26a inhibits H1N1 influenza A virus (IAV) replication may by binding to the conserved region of the PB1 gene^12^. However, another report suggests that miR-26a negatively regulates the production of IFN-β by targeting the 3` untranslated region (UTR) of the IFN-β gene^16^. Previous research shows that IFN-β protects both MARC-145 cells and porcine alveolar macrophages from PRRSV infection^17^, so miR-26a might promote PRRSV infection.

In the present study, we investigated the exact role of the overexpression miR-26a during PRRSV infection and the miRNAs studied in previous reports^9^,^12^ were used as controls. We found that miR-26a inhibits PRRSV replication in MARC-145 cells. Furthermore, this phenomenon was not related to miR-26a binding to the PRRSV genome but was the result of up-regulating the innate immune response. Our results are consistent with the emerging notion that miRNAs are broadly involved in viral infection of mammalian cells and suggest that miR-26a may represent a potential therapeutic target for antiviral intervention of PRRSV infection.

## Results

### miR-26a is a broad-range, anti-viral antagonist that is effective against PRRSV

**Figure 1:**
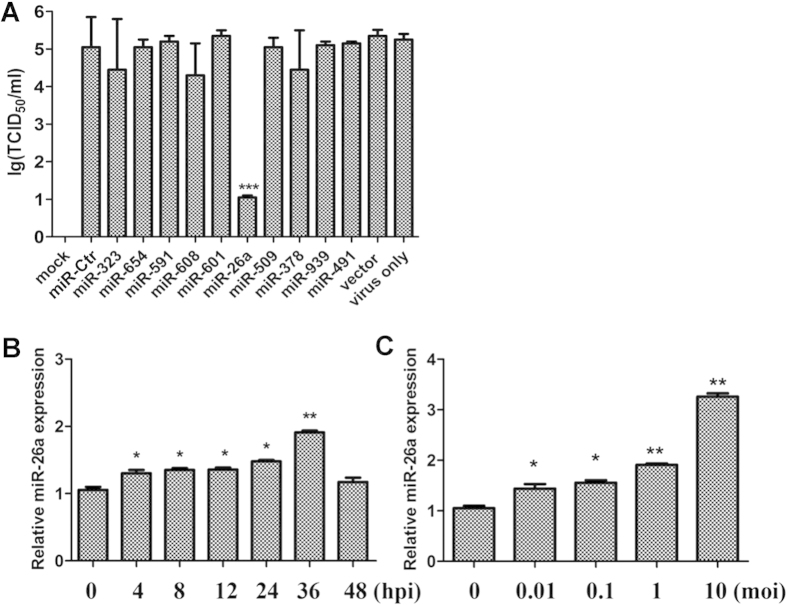
miR-26a is an antagonist of PRRSV. (**A**) MARC-145 cells were transfected with plasmids expressing miR-Ctr, miR-323, miR-654, miR-591, miR-608, miR-601, miR-26a, miR-509, miR-378, miR-939 and miR-491 or vector control (1 μg each). At 24 hpt, the cells were infected with VR2332 PRRSV at 0.1 MOI. The viral titers of the supernatants at 72 hpi were determined as the TCID_50_. (**B**) MARC-145 cells were infected with or without the PRRSV VR-2332 strain at 1 MOI for the indicated time. The expression of miR-26a was measured by q-PCR and normalized to the expression of miR-18 in each sample. (**C**) MARC-145 cells were infected with or without the PRRSV VR-2332 strain at the indicated MOIs for 36 h and miR-26a expression was measured as described in (**B**). These data are expressed as the mean ± SD and compared to the “virus only” (**A**) or “0 hpi” (**B** and **C**) groups by two-way ANOVA; * P < 0.5, ** P < 0.01 and *** P < 0.001.

First, miR-26a, miR-Ctr (the same nucleotides as miR-26a but randomized), miR-323, miR-654, miR-591, miR-608, miR-601, miR-509, miR-378, miR-939 and miR-491 were used to investigate the functions of miRNAs during PRRSV infection in susceptible cells (MARC-145). We found that only miR-26a suppresses PRSSV infection (Fig. 1A). In addition, after PRSSV infection, miR-26a expression (RNA level) significantly increased from 4 to 36 hpi (Fig. 1B). Further, PRRSV induced miR-26a expression in a dose-dependent manner (Fig. 1C). These data indicate that miR-26a is a PRRSV infection-responsive miRNA that can inhibit PRRSV replication in MARC-145 cells.

**Figure 2:**
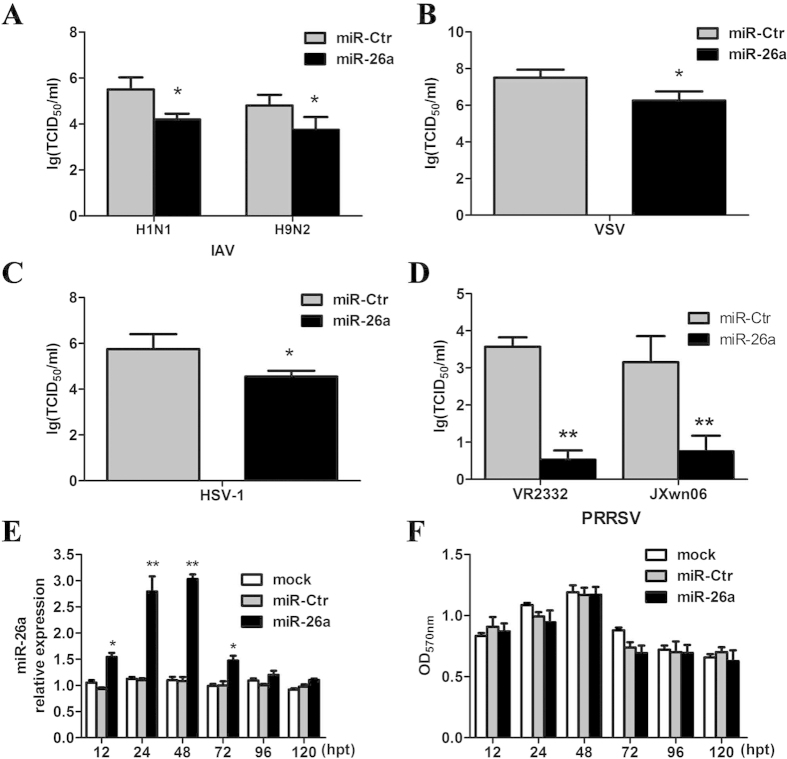
miR-26a is a broad-range antagonist of viral replication. MARC-145 cells were transfected with plasmids encoding miR-26a or control (miR-Ctr). Twenty-four hours post-transfection, the cells were infected with H1N1 and H9N2 IAV (**A**), VSV (**B**), HSV-1 (**C**), VR2332, and JXwn06 PRRSV (**D**) at a MOI = 1 for 72 h. The viral titers were determined as the TCID_50_. (**E**) The relative expression of miR-26a in MARC-145 cells transfected with miR-26a or miR-Ctr, as well as in mock-transfected cells, was measured by real-time PCR at the indicated times. (**F**) The viability of MARC-145 cells mock-transfected or transfected with miR-26a or miR-Ctr was determined by MTT assays up to 120 hpt. These data are expressed as the mean ± SD and compared to the miR-Ctr group (**A-D**) or mock group (**E-F**) by paired-sampled *t*-tests and two-way ANOVA; * P < 0.5, ** P < 0.01, and *** P < 0.001.

It was previously reported that miR-26a suppresses the replication of H1N1 (WSN) IAV^12^. To determine if miR-26a is a non-specific anti-viral agent, the inhibition of VSV, HSV-1 and PRRSV by miR-26a was investigated. Notably, these viral genomes do not contain putative miR-26a binding sites as defined by the MicroInspector program (data not shown). MARC-145 cells were transfected with 1 μg miR-26a and, 24 h later, infected with IAV (H1N1 or H9N2), VSV, HSV-1, or PRRSV (VR2332 or JXwn06) at a multiplicity of infection (MOI) of 0.1. The viral titers at 24 hpi were determined as the 50% tissue culture infective dose (TCID_50_); the transfection efficiency was >20%, as determined using pEGFP-N1 (data not shown). We found that miR-26a decreased the total viral titers of IAV, VSV and HSV-1 by approximately 10-fold (*p* < 0.05, Fig. 2A-C) and significantly inhibited the replication of PRRSV >100-fold (*p* < 0.001, Fig. 2D). These data suggest that miR-26a may act as a broad-spectrum viral antagonist, especially for PRRSV.

Additionally, the level of miR-26a in the transfected cells from 12-120 hpt was validated using real-time PCR. Transfection with plasmids expressing miR-26a resulted in a >50% increase in miR-26a levels in MARC-145 cells at 12 hpt, which peaked from 24–48 hpt (up to 3-fold) and gradually decreased to normal levels by 96 hpt (Fig. 2E). Next, MTT assays were used to determine if miR-26a affects cell viability. As shown in Fig. 2F, the viability of MARC-145 cells remained unchanged up to 120 hpt, regardless of the up-regulation of miR-26a expression levels.

### Dose-dependent and time-duration inhibition of viral replication by miR-26a

**Figure 3:**
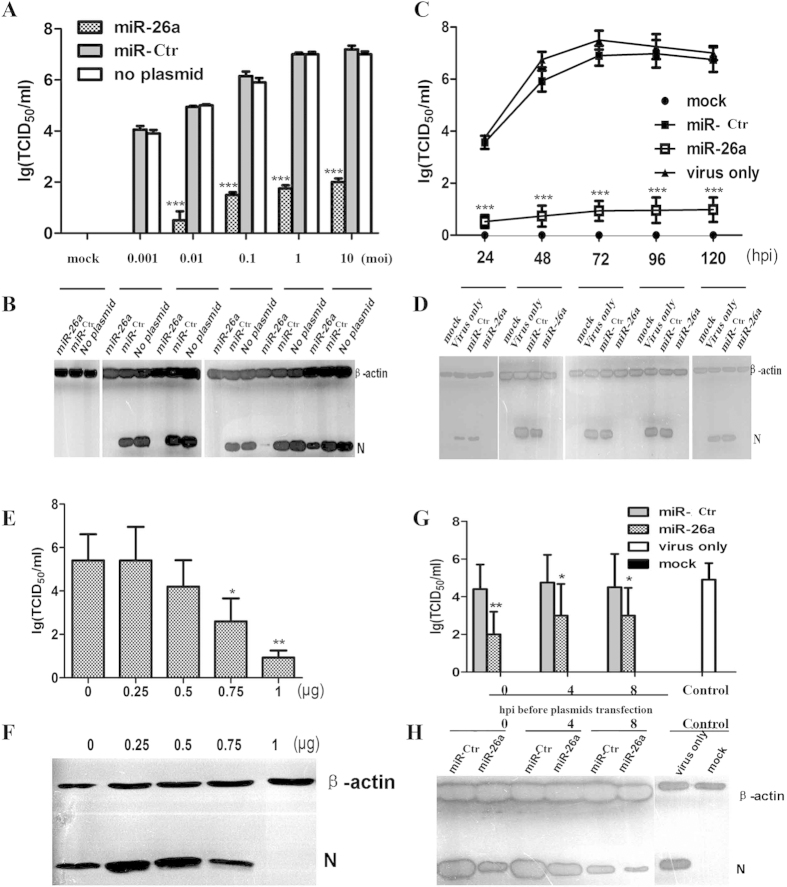
Inhibition of viral replication by miR-26a in MACR-145 cells was dose dependent and long lasting .**(A-B)** MARC-145 cells seeded in 24-well plates were first transfected with 1 μg plasmid (miR-26a or miR-Ctr) or mock transfected, followed by PRRSV VR-2332 infection at the indicated MOI or mock infection at 24 hpt. At 72 hpi, the culture supernatants were measured by TCID_50_ (**A**) and the cell lysates were analyzed with an anti-N polyclonal antibody by WB (**B**). (C-D) MARC-145 cells were first individually transfected with 1 μg plasmid expressing miR-26a or miR-Ctr or mock transfected, followed by PRRSV VR-2332 infection (MOI = 0.1) or mock infection at 24 hpt. At 24, 48, 72, 96, and 120 hpi, the culture supernatants were measured by TCID_50_ (**C**) and cell lysates were analyzed by WB (**D**). (E-F) MARC-145 cells seeded in 24-well plates were first transfected with the miR-26a or miR-Ctr plasmid in increasing amounts (0, 0.25, 0.5, 0.75, and 1 μg), followed by PRRSV VR-2332 infection (MOI = 0.1) at 24 hpt. At 48 hpi, the supernatants of the cell cultures were harvested and measured by TCID_50_ as the viral titers (**E**) and the cell lysates were analyzed by WB (**F**). (G-H) MARC-145 cells were first infected with 0.1 MOI PRRSV VR-2332, and 0, 4, or 8 h later, the cells were transfected with plasmid expressing miR-26a or miR-Ctr or mock transfected. At 48 hpi, the culture supernatants were measured by TCID_50_ (**G**) and the cell lysates were analyzed by WB (**H**). These data are expressed as the mean ± SD and compared to the miR-Ctr group (**A, C, G**) or “0 μg” group (**E**) by paired-sampled *t*-tests; * P < 0.5, ** P < 0.01, and *** P < 0.001.

The inhibitory effect of miR-26a described above was observed at a MOI of 0.1. Thus, we next investigated whether miR-26a displays the same suppression at lower and higher MOIs (0.001, 0.01, 0.1, 1 and 10) of PRRSV infection. MARC-145 cells were first transfected with 1 μg plasmid (miR-26a or miR-Ctr), followed by PRRSV VR2332 strain infection at 24 hpt. At 72 hpi, the cells were examined for the cytopathic effect (CPE), the culture supernatants were measured by TCID_50_, and cell lysates were analyzed by western blotting (WB). We found that the viral titers were significantly reduced by approximately 1,000- to 10,000-fold by miR-26a (Fig. 3A). Additionally, the levels of N protein were either undetectable at MOI = 0.001-0.1 or remarkably decreased at MOI = 1 and 10 (Fig. 3B). Further, in the presence of miR-26a, no CPE was induced by PRRSV at any MOI tested (Fig. S1).

To examine the duration of viral replication inhibition caused by miR-26a, MARC-145 cells were first transfected with 1 μg plasmid (miR-26a or miR-Ctr), followed by PRRSV infection (MOI = 0.1) at 24 hpt. At 24, 48, 72, 96, and 120 hpi, the culture supernatants were measured by TCID_50_, and cell lysates were analyzed by WB. As shown in Fig. 3C, compared to the control groups (virus only and miR-Ctr), miR-26a resulted in >100- to nearly a one million-fold reduction of virus yield during replication (24-120 hpi). Similarly, no CPE occurred until 96 hpi (data not shown). The same inhibition was also observed at the protein level, as measured by N protein in Fig. 3D. These results suggest that the inhibitory effect induced by miR-26a lasts for at least 120 hpi.

To measure the dose-dependent suppression of viral replication, MARC-145 cells seeded in 24-well plates were first individually transfected with the miR-26a or miR-Ctr plasmids in increasing amounts (0, 0.25, 0.5, 0.75, and 1 μg) and then infected with PRRSV (MOI = 0.1) at 24 hpt. At 48 hpi, the viral titers in the supernatants were measured by TCID_50_, and the cell lysates were analyzed by WB using an anti-N polyclonal antibody. We found that the cells transfected at a higher dose of miR-26a displayed a stronger antiviral effect, both at the virus titer and N protein expression levels (Fig. 3E-F).

To test if miR-26a could suppress PRRSV in previously infected cells, MARC-145 cells were infected with 0.1 MOI PRRSV, and 0, 4, or 8 h later, these cells were transfected with the miR-26a or miR-Ctr plasmids. At 48 hpi, cells were collected, and the viral titers were measured. As shown in Fig. 3G-H, miR-26a expression in pre-infected cells was also able to effectively inhibit viral replication. The efficiency of virus replication suppression induced by miR-26a at 4 or 8 h after viral infection was not different from that at 0 h after viral infection, and all of the associated viral titers were approximately 100-fold lower than that of the control groups (virus only and miR-Ctr).

### miR-26a suppresses the replication of PRRSV by impairing the synthesis of viral subgenomic mRNAs, and its inhibitor exacerbates PRRSV infection

We performed parallel assays in MARC-145 cells by transfecting either the miRNA expression vector (miR-26a or miR-Ctr) or miRNA inhibitor (anti-miR-26a or anti-control). At 24 hpt, the cells were inoculated with PRRSV at 0.1 MOI, and at 48 hpi, the culture supernatants and cells were harvested for further analysis (or 72 hpi, only for CPE observation).

**Figure 4:**
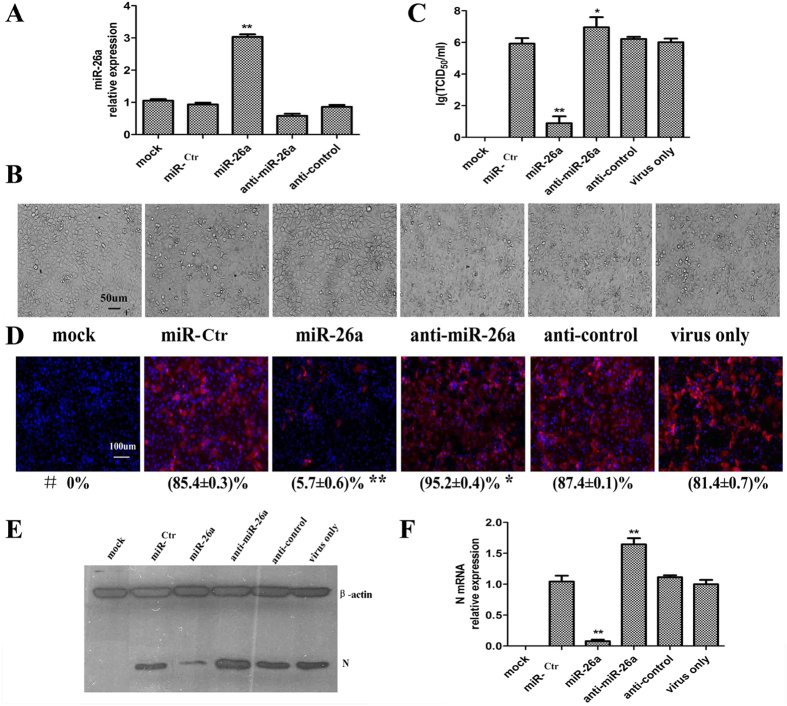
miR-26a suppressed the replication of PRRSV by impairing the synthesis of viral subgenomic mRNA, and a miR-26a inhibitor exacerbated PRRSV infection. MARC-145 cells were transfected with either the miRNA expression vector (miR-26a or miR-Ctr) or miRNA inhibitor (anti-miR-26a or anti-control) or mock transfected. Then, the cells were inoculated with PRRSV at 0.1 MOI or mock infected at 24 hpt. Five individual experiments (Exp. 1-5) were performed as followed: Exp. 1, at 24 hpt, the relative expression of miR-26a in MARC-145 cells was detected by real-time PCR (A); Exp. 2, at 72 hpi, CPE was observed (**B**), and the culture supernatants were harvested for TCID_50_ detection (**C**); Exp. 3-5, at 48 hpi, the N protein of PRRSV was detected by IFA (**D**) or WB (**E**) with an anti-N polyclonal antibody, and the mRNA of N was detected by real-time PCR (**F**). # indicates the efficiency of infection, expressed as the percent of N-positive cells relative to all cells from three independent experiments. These data are expressed as the mean ± SD and compared to the miR-Ctr group (**A, C, D** and **F**) by paired-sampled *t*-tests; * P < 0.5, ** P < 0.01 and *** P < 0.001.

First, quantitative real-time PCR (qRT-PCR) showed that at 24 hpt, miR-26a was highly expressed due to miR-26a plasmid transfection (miR-26a). The miR-26a expression level was not significantly decreased by miR-26a inhibitor transfection (anti-miR-26a) or in the other four controls (miR-Ctr, anti-Control, mock infection, and virus only) (Fig. 4A).

Second, at 72 hpi, the infected cells from the control groups (miR-Ctr, anti-Control, and virus only) became reticulated and detached from the monolayer. However, no CPE was observed in the miR-26a and mock groups, while CPE in the anti-miR-26a group was increased relative to the infected control groups (Fig. 4B).

Finally, at 48 hpi, the viral titers of the culture supernatants were measured as the TCID_50_ (Fig. 4C), the PRRSV N protein was detected by IFA (Fig. 4D) or WB (Fig. 4E) with anti-N polyclonal antibodies, and the N mRNAs were quantified by real-time PCR (Fig. 4F). Our results demonstrate that at the protein, RNA, and viral levels, the replication of PRRSV was significantly inhibited by the over-expression of miR-26a, and PRRSV replication was aggravated by knock-down of miR-26a via miR-26a inhibitor transfection.

### The genome of PRRSV is not the target of miR-26a

**Figure 5:**
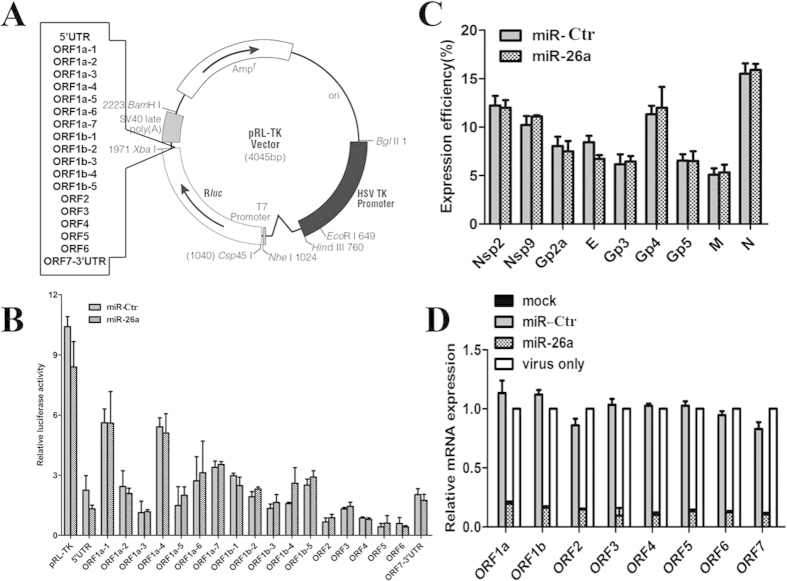
The genome of PRRSV was not the targe*t* of miR-26a . Fragments of the PRRSV genome were inserted into the 3`-UTR of the luciferase gene in the luciferase expression vector pRL-TK (Promega) (**A**). Then, co-expression of 1 μg miR-26a (or miR-Ctr) with the TK-ORFs in MARC-145 cells and luciferase activity assays were performed (**B**). (**C**) IFA was performed with an anti-myc monoclonal antibody to determine the expression efficiency in MARC-145 cells transfected with plasmids encoding 1 μg miRNAs (miR-26a or miR-Ctr) and the recombinant plasmids encoding the non-structural and structural proteins of PRRSV (including nsp2, nsp9, Gp2a, E, Gp3, Gp4, Gp5, M, and N) with a myc-tag at their N-terminus. (**D**) MARC-145 cells were first individually transfected with plasmids expressing miR-26a or miR-Ctr or mock transfected followed by PRRSV VR-2332 infection at 10 MOI or mock infection, and the mRNA level of each of the ORFs in the PRRSV genome was measured by real-time PCR at 6 hpi.

To determine if miR-26a directly targets the PRRSV genome and/or regulates host genes, we first used two algorithms (PicTar and TargetScan Release 5.1) to predict the PRRSV gene targets of miR-26a. We found that the seed sequence of miR-26a was partially complementary to each of the PRRSV subgenomic sequences. Thus, each PRRSV ORF was inserted between the 3` end of the luciferase gene and the 5’ end of the poly(A) tail in the luciferase expression vector pRL-TK (Promega) (Fig. 5A). MARC-145 cells were then co-transfected with miR-26a (or miR-Ctr) and TK-ORFs, and luciferase activity assays were performed. As shown in Fig. 5B, miR-26a did not target the genome of PRRSV. To confirm this result, recombinant plasmids encoding the non-structural and structural proteins of PRRSV (including nsp2, nsp9, Gp2a, E, Gp3, Gp4, Gp5, M and N) with a myc-tag at their N-terminus were co-transfected with either miR-26a or miR-Ctr, and IFA with an anti-myc monoclonal antibody was performed to determine if miR-26a inhibited the production of each protein. Figure 5C shows that ectopic expression of miR-26a did not reduce the fluorescence intensity.

To further confirm this relationship, we used real-time RT-PCR to determine the effect of miR-26a on the mRNA levels of PRRSV during the early stage of viral infection (6 hpi at MOI = 10; the primer sequences are available from the corresponding author upon request). When miR-26a was over-expressed, the levels of each mRNA in the subgenome were dramatically decreased (Fig. 5D). This indicates that miR-26a inhibited viral infection by regulating host genes that are involved in the control of PRRSV replication.

### miR-26a triggers the INF signaling pathway

**Figure 6:**
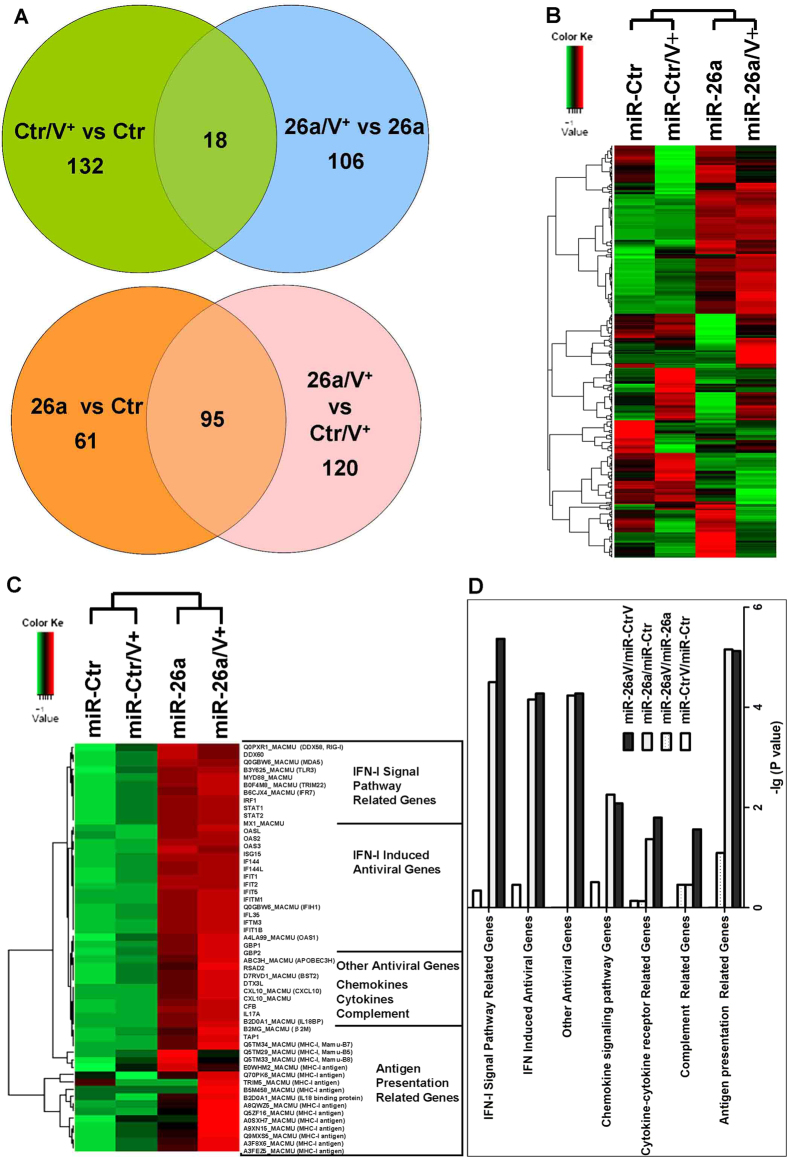
INF responses were triggered by miR-26a . MARC-145 cells were transfected with miR-26a or miR-Ctr for 24 h and then infected with PRRSV (VR2332) or mock infected. Cells were harvested 24 hpi, and total cellular RNA was analyzed by RNA-seq. (A) Venn diagrams of all DEGs between the four groups. (**B**) Heat-maps indicating the fold-changes are shown as matrices with rows representing genes and columns representing the groups with miR-26a/miR-Ctr transfection and PRRSV/MOCK infection. Red represents up-regulation, and blue represents down-regulation. (**C**) Heat-maps indicating the defined distinct functional clusters that were associated with distinct functional annotations. (**D**) Enrichment analysis results of GO ‘Biological Process’ terms and KEGG pathways are shown for the clusters in (**C**) with the most significance (p < 0.05). Categories are displayed in the graphs as bars, sorted from bottom (most significant) to top.

To investigate the antiviral mechanism of miR-26a, RNA-seq was used to determine the changes in cellular transcript levels after miR-26a or miR-Ctr treatment with or without PRRSV infection. The samples were divided into four groups: miR-Ctr, miR-Ctr/V, miR-26a, and miR-26a/V (where “V” stands for PRRSV infection). Sixteen billion tags were obtained from sequencing (four billion for each sample). Using the Cuffdiff program, the numbers of differentially expressed genes (DEGs) between any two groups are shown in Table 1. Of the total transcripts, 156 were significant hits in the miR-26a-treated library, and 215 were significant in the PRRSV-infected plus miR-26a-transfected library. Ninety-five were found in both of the libraries (Fig. 6A). In addition, 150 DEGs were found after PRRSV infection in MARC-145 cells, 124 DEGs were significant after virus infection in the miR-26a transfected cells, but only 18 hits were found in both libraries (Fig. 6A).

To uncover functional characteristics of the DEGs among the four groups, we performed an enrichment analysis of Gene Ontology (GO) terms (biological process) and KEGG pathways (Fig. 6B-D). Interestingly, two clusters were associated with distinct functional terms, including the IFN signaling pathway and the antigen presentation pathway (Fig. 6C-D, Table 2). Heat-maps indicate that both the IFN signaling pathway and antigen presentation pathway were triggered by miR-26a and then enhanced by PRRSV infection (Fig. 6C). Some IFN-stimulated genes were significantly up-regulated by miR-26a, including MX1, IFI44/IFI44L, the OAS family, and the IFIT family. The expression of other antiviral genes, such as RSAD2 and BST2, were also enhanced by miR-26a. Moreover, the expression of several chemokines (CXCL10), cytokines (IL-17A and IL28BP), and complements (CFB) was also increased.

## Discussion

miRNAs are crucial post-transcriptional regulators of many biological systems, including mammalian immune systems^18^, which function as host defenses against both RNA and DNA viruses. Together, the host- or virus-encoded miRNAs and their target genes form novel regulatory networks between the host and the virus^8^,^9^,^10^,^11^,^12^,^13^,^14^,^15^. In the present study, we demonstrated that miR-26a is an effective antagonist against several RNA viruses (including IAV, VSV, and PRRSV) and a DNA virus (HSV-1). Transfection of a miR-26a-expression plasmid before and after PRRSV infection significantly inhibited the replication of PRRSV in cells.

Many recent studies indicate that miR-26a is involved in physiological and pathological processes such as proliferation, megakaryocytopoiesis, innate immunity, neurodegenerative diseases, and tumorigenesis. For instance, miR-26a functionally antagonizes human breast carcinogenesis by targeting MTDH and EZH2 (a component of the Polycomb repressive complex 2)^19^. Kota *et al.* (2009) report that delivery of miR-26a results in dramatic protection from hepatocellular carcinoma (HCC) by targeting cyclins D2 and E2^20^. Salvatori *et al.* (2011) find that expression of miR-26a in acute myeloid leukemia cells inhibits cell cycle progression by down-regulating cyclin E2 expression, and they highlight miR-26a as an attractive therapeutic target in leukemia^21^. Through targeting the EZH2 gene and repressing the expression of EZH2, miR-26a dramatically suppresses cell proliferation and colony formation by inducing G(1)-phase cell cycle arrest and inhibits tumorigenesis of nasopharyngeal carcinoma^22^. Further, miR-26a1/2 and miR-26b down-regulate BDNF (brain-derived neurotrophic factor) expression, which is a neurotrophin that plays an essential role in neuronal development and plasticity^23^. In the process of gliomagenesis, miR-26a is a direct regulator of PTEN (phosphatase and tensin homolog), which is involved in the Akt pathway, and highlights dysregulation of Akt signaling as crucial to the development of glioma^24^. Ectopic expression of miR-26a influences cell cycle progression by targeting the *bona fide* oncogene EZH2, a polycomb protein and global regulator of gene expression that was not previously known to be regulated by miRNAs^25^. Finally, osteogenic differentiation of human adipose tissue-derived stem cells is modulated by miR-26a targeting of the SMAD1 transcription factor^26^.

In this report, we found that a miR-26a-mediated antiviral mechanism is utilized by host cells to defend against PRRSV infection. However, whether the targets of miR-26a (including BDNF, cyclin E2, EZH2, MTDH, PTEN, and SMAD1) are involved the replication of PRRSV remains unknown. Thus, our future work will focus on the identification of targets of miR-26a.

In elucidating the mechanism of miR-26a antiviral activity, luciferase reporter assays and IFA were used to determine if the viral genome is the target of miRNA-26a. The prevailing wisdom is that if the sequences of the miRNA and its mRNA targets are perfectly matched, the target mRNA is degraded. However, repression of translation is the most common effect if the seed sequences of the miRNA and the target mRNA only partially match one another. The subgenomic genes of VR-2332 PRRSV, including the 3` and 5` UTRs, were directionally cloned into the 3`-UTR of the luciferase gene in the pRL-TK vector (Fig. 5A). If miR-26a targeted the genome of PRRSV, luciferase activity would decrease. However, our results demonstrate that ectopic expression of miR-26a did not alter luciferase activity, indicating that miR-26a did not target the PRRSV genome (Fig. 5B). In addition, during the early stage of PRRSV infection (6 hpi), the synthesis of all viral subgenomic mRNAs was impaired by miR-26a, which corresponded to the virus titers measured after miR-26a transfection (Fig. 3). These results indicated that some cellular transcription factor(s) participating in PRRSV transcription and/or replication are regulated by miR-26a (Fig. 5D).

Similar to our results, Li *et al.* (2015) also prove that miR-26a suppresses PRRSV replication in cells using a synthesized miR-26a mimic instead of a plasmid-based expression system. Furthermore, their luciferase reporters show that miR-26a does not directly target the PRRSV genome but instead affects the expression of type I INF and the IFN-stimulated genes MX1 and ISG15 during PRRSV infection^27^.

To systematically study the anti-PRRSV infection mechanism of miR-26a, host cell transcripts were analyzed by RNA-seq after transfecting miR-26a with or without PRRSV infection in MARC-145 cells. The results showed that the MX1 and ISG15 genes are only two of the significant number of factors involved in regulating anti-viral responses, including the RIG-I- and TLR-mediated IFN signaling pathways (Fig. 6C, Table 2). The transcripts of RIG-I, MDA5, IRF7 and TLR3 were also significantly up-regulated by miR-26a (Table 2). In addition, many ISGs were activated by miR-26a, including OASL, OAS1, OSA2, OAS3, MX1 and ISG15, which have high antiviral activity^28^. Although miR-26a activated the RIG-I/MDA5 and TLR3/MYD88 IFN signaling pathways, the expression of IFN-β was not increased in this study, which differs from the results of luciferase reporters^27^. The most probable reason for this phenomenon is that miR-26a may target the 3`-UTR of the IFN-β gene and mediate its mRNA degradation^16^. These results provide further evidence that miR-26a could negatively regulate PRRSV replication by enhancing the innate immune response. Thus, cellular conditions and the transfection efficiency of plasmids expressing miR-26a may influence the antiviral function. Further, some differences in virus titers after miR-26a transfection were observed in Fig. 3A,3C,2D, as well as in the results of WB detection for PRRSV N protein in Fig. 3, though the antiviral functions of mi-R26a were significant in those experiments.

Vaccination and drugs are the principal means used to control and treat PRRSV infection. An array of PRRS vaccines have been developed, but none provide sustainable disease control because they suffer from both immune suppression and evasion strategies of the virus, as well as the antigenic heterogeneity of field strains. Effective drugs would be another useful strategy to control this disease. INFs, including type I (IFN-α, IFN-β) and type III (IFN-λ), are widely studied for their antiviral effects against PRRSV^17^,^29^. Further, Wei *et al.* (2011) explore the inhibitory effects of indigowoad root polysaccharides on PRRSV replication in MARC-145 cells^30^.

Unfortunately, there is currently no effective drug that can be used to treat PRRSV infection. Hence, it is imperative to develop other antiviral strategies to combat PRRSV infection, such as RNA interference (RNAi). Previous research demonstrates that PRRSV replication is inhibited by RNAi induced by recombinant plasmids or adenoviruses expressing shRNAs targeting viral subgenomes^31^,^32^,^33^,^34^,^35^. Recently, Xiao *et al.* (2011) were the first to attempt to suppress HP-PRRSV replication in MARC-145 cells through vector-based and lentiviral-mediated artificial microRNAs targeting the GP5 or M protein coding sequences of PRRSV^36^. Guo *et al.* (2013) report that miR-181 directly impairs PRRSV infection by specifically binding to ORF4 of the viral genomic RNA^8^.

Here, we aimed to investigate if any cellular miRNA(s) could persistently inhibit PRRSV replication. We found that cellular miR-26a significantly inhibits the replication of the highly pathogenic JXwn06 strain, as well as the traditional VR2332 strain, up to 120 hpi in a dose-dependent manner (Fig. 2), with evidence of alleviating CPE (Fig. 4 and Fig. S1), reducing the viral titer in cell supernatants (Fig. 1, 2, 3, 4), and inhibition of viral RNA and protein synthesis (Fig. 3, 4, 5). PRRSV can persist in pigs for long periods of time after the initial infection, and persistently infected animals can shed infectious virus for several months^37^. To address whether miR-26a could counteract virus replication in infected cells, MARC-145 cells were transfected with miR-26a 0, 4, and 8 hpi with PRRSV VR2332. Our results show that the viral titers were reduced by approximately 2 logs of TCID_50_ in each experimental group transfected with miR-26a (Fig. 3G-H), suggesting that miR-26a might be applied as an effective anti-PRRSV strategy during PRRS outbreaks.

Overall, we provide evidence for an intricate interplay between a cellular miRNA and host antiviral responses against PRRSV infection. Host-encoded miR-26a had an antiviral effect on PRRSV replication via induction of the innate INF response. Our results implicate that cellular miR-26a may be used as a potential therapeutic strategy against PRRSV infection in the future.

## Methods

### Cells and viruses

MARC-145 cells were maintained in Dulbecco’s modified Eagle’s medium (DMEM, GIBCO) supplemented with 10% heat-inactivated fetal bovine serum (FBS, GIBCO) at 37 °C in 5% CO_2_ atmosphere. MARC-145 cells were used to propagate and titer the VR-2332 PRRSV strain (GenBank No. PRU87392), the highly pathogenic PRRSV JXwn06 strain (GenBank No. EF6410081, a kind gift of Hanchun Yang, China Agricultural University), H1N1 (the A/WSN/1933 (H1N1) strain, which was rescued from cDNAs^38^), H9N2 (the A/chicken/Shandong/BD/2008 strain^39^) IAV, VSV (Indiana strain^40^,), and type I herpes simplex virus (HSV-1, a kind gift of Chunfu Zheng, Wuhan Virology Institute^41^,).

### Reagents and antibodies

Mouse anti-β actin monoclonal antibody and TRITC- and HRP-conjugated anti-mouse IgGs were purchased from Kangwei Biotech (Beijing, China). Mouse anti-His and anti-myc monoclonal antibodies were purchased from Santa Cruz (CA, USA). The mouse anti-N polyclonal antibodies were developed by our laboratory.

### Plasmid construction

The luciferase expression vector pRL-TK (Promega) was used as the parent vector for 3`-UTR reporter analysis experiments. The genome of VR-2332 PRRSV was divided into 22 fragments with 25-bp overlapping flanking regions between each fragment. Then, these fragments were directionally cloned into the 3`-UTR of the luciferase gene in the pRL-TK vector (as shown in Fig. 5A). These vectors were named pRL-TK-5`UTR, pRL-TK-ORF1a-1, pRL-TK-ORF1a-2, pRL-TK-ORF1a-3, pRL-TK-ORF1a-4, pRL-TK-ORF1a-5, pRL-TK-ORF1a-6, pRL-TK-ORF1a-7, pRL-TK-ORF1b-1, pRL-TK-ORF1b-2, pRL-TK-ORF1b-3, pRL-TK-ORF1b-4, pRL-TK-ORF1b-5, pRL-TK-ORF2, pRL-TK-ORF3, pRL-TK-ORF4, pRL-TK-ORF5, pRL-TK-ORF6, pRL-TK-ORF6 and pRLTK-ORF7-3’UTR. To facilitate cloning, an *Xba*I restriction site was added to the 5`-primer and the 3`-primer. All inserts were sequenced to verify polymerase fidelity. The primer sequences are available from the corresponding author upon request.

The microRNA expression plasmids miR-26a, miR-654, miR-591, miR-601, miR-608, miR-654, miR-509, miR-378, miR-939, miR-491 and miR-Ctr (the same nucleotides as miR-26a but randomized) were kind gifts of Professor Wenlin Huang from our institute^9^.

### Plasmid transfection and virus infection

The plasmids and miRNA inhibitors were transfected into cells using Lipofectamine^TM^2000 (Invitrogen) according to the manufacturer’s recommendations. The GFP expression plasmid pEGFP-N1 was used as an internal control. Twenty-four hours post-transfection, the cells were washed with PBS three times and infected with virus in infection medium (fresh DMEM with 2% FBS, except for IAV, which required fresh DMEM lacking FBS and containing 2.5 g/ml trypsin) at the indicated MOI. After incubation for 1 h, the cells were washed three times with PBS, and medium was added to the cells.

### Indirect immunofluorescence assay (IFA)

MARC-145 cells seeded in 24-well plates were transfected with miR-26a and control (miR-Ctr) plasmids for 24 h and then infected with VR2332 PRRSV at MOI = 0.1. At 48 h post-infection, MARC-145 cells were washed with PBS, fixed in 4% paraformaldehyde, and permeabilized with PBST (0.1% Triton X-100 in PBS). Then, they were incubated for 1 h at 37 °C with 4% BSA in PBS followed by incubated with a polyclonal antibody against the N protein (1:500 mouse sera) for 1 h at 37 °C. After washing five times with PBST, the cells were incubated for 1 h at 37 °C with TRITC-conjugated anti-mouse IgG (1:100). After washing five times with PBST, the cells were stained with DAPI (4`,6-diamidino-2-phenylindole) and observed under a Leica confocal microscope.

### Western blotting (WB) analysis

WB was performed as previously described^9^. PVDF membranes were probed with a 1:2,000 dilution of mouse anti-N and/or anti-GP5 polyclonal antibody. To normalize protein loading, the PVDF membranes were simultaneously incubated with mouse anti-β actin monoclonal antibody at a dilution of 1:3,000. HRP-conjugated goat anti-mouse IgG (1:5,000) was used as a secondary antibody.

### Virus titration

MARC-145 cells were trypsinized and seeded in 96-well plates 24 h before virus infection. Viral supernatants were 10-fold serially diluted with infection medium and added to each well with eight technical replicates of each dilution. One to five days (based on the indicated types of virus) after infection, the TCID_50_ was calculated by the Reed-Muench method^42^.

### RNA quantification by real-time PCR

Total RNA was extracted with TRIzol reagent (Invitrogen) following the manufacturer’s instructions. Real-time quantitative RT-PCR analysis was performed using a LightCycler (Roche) and SYBR RT-PCR kits (Takara). Primer sequences for the PRRSV protein coding genes and GADPH (internal control) are available from the corresponding author upon request. The relative expression level of the indicated genes was normalized to that of the GADPH internal control using the 2^−ΔΔCt^ cycle threshold method.

For miRNA analysis, small RNAs including miRNA were isolated using a mirVana™ miRNA Isolation Kit (Applied Biosystems). The expression of miR-26a was analyzed using specific primers and probes (Applied Biosystems). The relative expression levels of the miRNAs were normalized to that of the miR-18 internal control using the 2^−ΔΔCt^ cycle threshold method.

### Dual luciferase reporter assays

Luciferase assays were performed using a dual-luciferase reporter assay system kit (Promega) according to the manufacturer’s protocol. Collected cells were washed once with cold PBS, and passive lysis buffer (100 μl) was then added to the cells. After 10 min, the supernatants were collected by centrifugation at 12,000 g for 30 s, and the relative luciferase expression values were analyzed using a Modulus single-tube multimode reader (Promega). The relative luciferase expression was calculated as the expression of Renilla luciferase (pRL-TK) divided by the expression of firefly luciferase (pGL3-control vector).

### RNA-seq analysis

Following transfection with miRNA-26a or miR-Ctr, MARC-145 cells were mock infected or infected with PRRSV VR2332 at MOI = 2 and then harvested 24 hpi. Total cellular RNA was prepared according to the manufacture’s protocol using an RNA Isolation Kit (Invitrogen). All of the RNA Integrity Number (RIN) values were >7.0, and the 28S:18S rRNA ratios were >1.9, as confirmed using an Agilent Bioanalyzer.

cDNA libraries were constructed from poly(A)-enriched RNA using Illumina kits and sequenced by 2*100 paired-end sequencing on an Illumina HiSeq 2000 instrument. The FASTQ read files for the four samples were used for further data analysis. Data for gene counts were obtained using the Mayo Clinic pipeline and Burrows-Wheeler Alignment (BWA). To analyze the Illumina reads, TopHat and Cufflinks were used to investigate the DEG profiles and changes in transcript abundance in the miR-26a- or miR-Ctr-transfected MARC-145 cells with or without PRRSV infection. Four files of transcriptome data from the miR-Ctr-, miR-Ctr/V-, miR-26a- and miR-26a/V-inoculated groups were aligned to the UCSC Rhesus Macaque genome build in preparation for differential expression analysis. The four files were processed through Cufflinks to assemble the aligned RNA-seq reads into transcripts and estimate the abundances in FPKM of the paired–end reads. The Cufflinks q-value was the false discovery rate (FDR)-adjusted p-value of the uncorrected test statistic; the q-value used in this study was 0.05. The significance status was “yes” when p > q after Benjamini-Hochberg correction for multiple-testing. Cuffmerge was then used to create a single transcript dataset from the multiple reconstructions. Two runs were then conducted using the miR-26a *vs.* miR-26a/V and the miR-Ctr *vs.* miR-Ctr/V datasets and the Cuffdiff program to test for differential expression and regulation among the two disease states. Gene annotation of all significant hits was then performed using a MySQL database matching to the Ensembl Sscrofa 9.56 reference genome currently supported by the Integrative Genomics Viewer (Broad Institute). Finally, heat-maps were analyzed using Cluster and TreeView-1.1.6r2-win software.

### Statistical analyses

Statistically significant differences between experimental groups were determined using Student’s *t*-test and analysis of variance (ANOVA) with the GraphPad Prism software package (GraphPad Software Inc., La Jolla, CA, USA). P-values <0.05 were considered statistically significant.

## Additional Information

**How to cite this article**: Jia, X. *et al.* Cellular microRNA miR-26a suppresses replication of porcine reproductive and respiratory syndrome virus by activating innate antiviral immunity. *Sci. Rep.* **5**, 10651; doi: 10.1038/srep10651 (2015).

## Supplementary Material

Supporting Information

## Figures and Tables

**Table 1:** Table of transcript counts.

**Compared groups**	**Significant hits**	**Up-regulated genes**		**Down-regulated genes**	
		**Total**	**>4-fold**	**Total**	**>4-fold**
aCtr/V+ *vs.*^b^Ctr	150	53	11	97	14
c26a/V+ *vs.*^d^26a	124	76	12	48	10
26a *vs.*Ctr	156	114	17	42	14
26a/V+*vs.*Ctr/V+	215	147	22	68	14

^a^Ctr/V+, Samples from the miR-Ctr transfected MARC-145 cells and then infected by PRRSV.

^b^Ctr, Samples from the miR-Ctr transfected MARC-145 cells without PRRSV infection.

^c^26a/V+, Samples from the miR-26a transfected MARC-145 cells and then infected by PRRSV.

^d^26a, Samples from the miR-26a transfected MARC-145 cells without PRRSV infection.

**Table 2:** Significantly differently expressed antiviral-related genes.

**Gene_id**	**Gene name**	**Protein name**	**Reads value**			
			**miR-Ctr**	**miR-Ctr /V+**	**miR-26a**	**miR-26a /V+**
ENSMMUG 00000012442	Q0PXR1_MACMU	RIG-I	39.936	56.1769	96.2302	105.098
ENSMMUG 00000001880	DDX60	RNA helicase	7.11089	9.57459	18.8027	23.9935
ENSMMUG 00000003202	Q0GBW6_MACMU	MDA5	8.13121	9.7414	20.8046	21.4849
ENSMMUG 00000021762	B3Y625_MACMU	TLR3	3.63154	4.17816	5.89225	6.50073
ENSMMUG 00000000995	MYD88_MACMU	MYD88	21.0263	23.8942	30.0516	33.2777
ENSMMUG 00000000338	B0F4M8_MACMU	TRIM22	18.1293	20.0728	45.7446	45.3348
ENSMMUG 00000004014	B6CJX4_MACMU	IRF7	15.4549	19.3094	27.0761	32.8857
ENSMMUG 00000008100	IRF1	IRF1	15.7938	15.4862	18.3734	22.2204
ENSMMUG 00000005613	STAT1	STAT1	300.529	336.546	449.421	458.023
ENSMMUG 00000000803	STAT2	STAT2	49.4468	60.3664	81.3158	94.7481
ENSMMUG 00000015329	MX1_MACMU	MX1	2.18566	2.15638	9.75438	11.3951
ENSMMUG 00000006441	OASL	OASL	4.60616	5.75103	32.5027	33.5457
ENSMMUG 00000012782	A4LA99_MACMU	OAS1	50.0421	57.5138	120.899	121.939
ENSMMUG 00000008505	OAS2	OAS2	13.3652	16.8814	71.096	77.2071
ENSMMUG 00000002674	OAS3	OAS3	11.0961	15.7502	21.3736	27.3624
ENSMMUG 00000001819	ISG15	ISG15	364.697	411.249	696.742	724.377
ENSMMUG 00000002350	IFI44	IFI44	40.7338	50.3358	97.2388	100.364
ENSMMUG 00000001569	IFI44L	IFI44L	37.5965	46.8464	81.6844	88.4203
ENSMMUG 00000008524	IFIT1B	IFIT1B	0.10658	0.347099	2.07998	0.7615
ENSMMUG 00000017533	IFIT1	IFIT1	95.4837	133.165	241.969	249.105
ENSMMUG 00000004185	IFIT2	IFIT2	16.6212	22.9426	55.0714	55.1577
ENSMMUG 00000017534	IFIT5	IFIT5	8.94529	10.8581	17.5394	18.817
ENSMMUG 00000013257	IFITM1	IFITM1	1370.41	1352.74	2098.44	2379.24
ENSMMUG 00000003202	Q0GBW6_MACMU	IFIH1	8.13121	9.7414	20.8046	21.4849
ENSMMUG 00000015999	IFI35	IFI35	213.129	217.295	303.023	320.216
ENSMMUG 00000029345	IFITM3	IFITM3	243.185	223.868	403.005	306.803
ENSMMUG 00000000662	GBP1	GBP1	5.78555	6.50819	15.5999	17.89
ENSMMUG 00000000663	GBP2	GBP2	17.1539	20.9365	27.9864	30.9183
ENSMMUG 00000001016	ABC3H _MACMU	APOB EC3H	18.3777	22.3224	35.7164	33.1611
ENSMMUG 00000015969	RSAD2		1.06702	1.16405	8.96142	9.62481
ENSMMUG 00000005829	D7RVD1 _MACMU	BST2	141.61	147.901	206.448	214.383
ENSMMUG 00000016383	DTX3L	E3 Ub- ligase	17.4219	21.153	31.859	30.6322
ENSMMUG 00000020903	CXL10_MACMU	CXCL10	13.6898	18.9237	52.6015	57.0937
ENSMMUG 00000000731	CFB	CFB	35.0347	41.0643	46.4513	59.6524
ENSMMUG 00000007403	IL17A	IL17A	0	0	0	3.00448
ENSMMUG 00000007340	B2D0A1_MACMU	IL19BP	9.67079	8.55637	15.9678	16.343
ENSMMUG 00000003760	B2MG_MACMU	β2M	889.55	929.665	1502.23	1434.64
ENSMMUG 00000029790	TAP1	TAP1	20.8527	23.4007	35.9497	40.611
ENSMMUG 00000000335	TRIM5_MACMU	TRIM5	16.0166	19.0978	29.5425	30.2378
ENSMMUG 00000003765	Q5TM34_MACMU	Mamu- B7	43.7021	44.2717	57.1338	64.1765
ENSMMUG 00000030622	Q5TM29_MACMU	Mamu- B5	8.63914	12.4651	15.7224	13.5089
ENSMMUG 00000002570	Q5TM33_MACMU	Mamu- B8	14.3725	18.6914	25.8013	24.2268
ENSMMUG 00000029841	E0WHM2_MACMU	MHC-I Ag	65.0841	78.1137	97.9884	100.132
ENSMMUG 00000030910	Q70PK6_MACMU	MHC-I Ag	25.6322	28.6997	39.5796	40.7759
ENSMMUG 00000030281	B5M458_MACMU	MHC-I Ag	72.1632	68.4805	113.045	118.437
ENSMMUG 00000016091	A8QWZ5_MACMU	MHC-I Ag^a^	66.2566	70.0493	99.9797	119.412
ENSMMUG 00000031913	Q5ZF16_MACMU	MHC-I Ag	34.5186	35.689	50.8139	57.79
ENSMMUG 00000019387	A0SXH7_MACMU	MHC-I Ag	21.5155	21.672	32.5367	33.5981
ENSMMUG 00000029875	A9XN15_MACMU	MHC-I Ag	41.5605	41.7899	59.3609	65.6841
ENSMMUG 00000029555	Q9MXS5_MACMU	MHC-I Ag	119.448	74.8487	128.766	154.922
ENSMMUG 00000030234	A3F8×6_MACMU	MHC-I Ag	7.92415	6.34866	9.7085	12.1832
ENSMMUG 00000029561	A3FEZ5_MACMU	MHC-I Ag	39.1468	39.8374	55.7683	75.5751

^a^Ag, antigen.
