# Supplementary material for: Cellular microRNA miR-26a suppresses replication of porcine reproductive and respiratory syndrome virus by activating innate antiviral immunity
Source: Sci Rep. 2015 May 27;5:10651. doi: 10.1038/srep10651 (PMC4445041; doi:10.1038/srep10651)
Supplement: Supporting Information [file srep10651-s1.pdf]

Cellular microRNA miR-26a suppresses replication of porcine reproductive and respiratory syndrome virus by activating innate antiviral immunity

Xiaojuan Jia [1,2,†], Yuhai Bi [2,†], Jing Li [2], Qing Xie [4], Hanchun Yang [1*], Wenjun Liu [2,3,*]

[1] Key Laboratory of Animal Epidemiology and Zoonosis of Ministry of Agriculture, College of Veterinary Medicine and State Key Laboratory of Agrobiotechnology, China Agricultural University, Beijing 100193, China.

[2] CAS Key Laboratory of Pathogenic Microbiology and Immunology, Institute of Microbiology, Chinese Academy of Sciences, Beijing 100101, China.

[3] University of Chinese Academy of Sciences, Beijing 100101, China.

[4] Department of Clinical Laboratory, Beijing Shijitan Hospital, Capital Medical University, Beijing 100038, China.

† Theses authors contributed equally to this work.

*Address correspondence to Wenjun Liu, liuwj@im.ac.cn, or Hanchun Yang, yanghanchun1@cau.edu.cn.

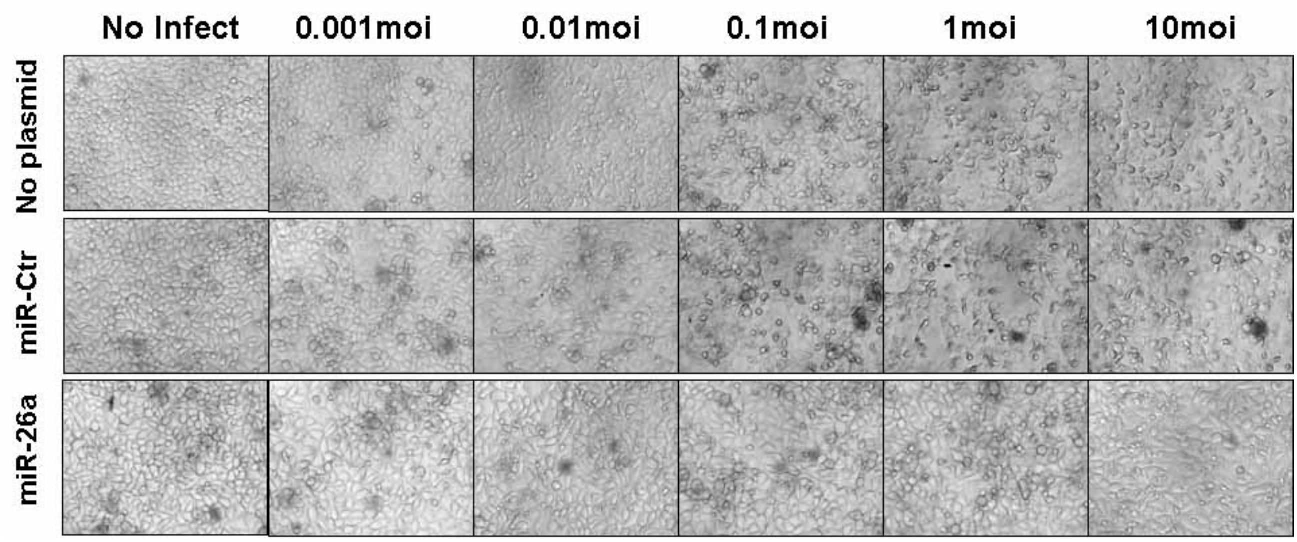

Supplementary Figure S1. miR-26a can inhibit CPE caused by PRRSV in MARC-145 cells.

MARC-145 cells were transfected with 1 μg plasmid (miR-26a or miR-Ctr) and then infected with PRRSV VR2332 at 24 hpt. At 72 hpi, the cells were examined for the cytopathic effect (CPE).
